# Supplementary material for: Poultry-associated nitrofurantoin-resistant and pre-resistant Escherichia coli clones are found in multiple countries and one-health compartments
Source: One Health. 2025 Oct 13;21:101241. doi: 10.1016/j.onehlt.2025.101241 (PMC12554031; doi:10.1016/j.onehlt.2025.101241)
Supplement: Supplementary file 1 — Supplementary material: Supplementary Tables S1-S8 [file mmc1.pdf]

Supplementary information for:

Poultry-Associated Nitrofurantoin-Resistant and Pre-Resistant Escherichia coli Clones are Found in Multiple Countries and One-Health Compartments

Jordan E Sealey, Beth Astley, Oliver Mounsey & Matthew B Avison*

University of Bristol, Bristol, UK

*Correspondence to Matthew B. Avison, University of Bristol, School of Cellular & Molecular Medicine, Biomedical Sciences Building, University Walk, Bristol. BS8 1TD, UK. Tel:+441173312036. Email: bimba@bristol.ac.uk

Table S1 Reference genomes used for sequencing alignment

ST	*E. coli* reference genome accession
ST69	SAMN40242201
ST665	SAMN25849693
ST919	SAMN11444779
ST752	SAMN32868256
ST6805	SAMEA112328101
ST7529	SAMN28864606

Table S2. Practices common to both dogs excreting NFT-R *E. coli* from the city of Bristol as reported by owners in a survey compared with how common each practice is among the canine cohort (n=297) in this study

Practice	Positive for practice – NFT-R excreters	Positive for practice – Not NFT-R excreters	Fisher's exact (p=)
Often/very often exercising on roads/streets	2/297	191/297	0.54
Often/very often exercising in parks	2/297	284/297	1
Sometimes exercising on beaches	2/297	195/297	0.5
Sharing a home with a cat	2/297	48/297	0.028
Fed raw meat	2/297	29/297	0.011

Practices positive for one NFT-R excreting dog only: walking in the countryside (not around livestock); swimming in lakes; swimming in ponds; swimming in rivers; received antibiotics in the last 6 months. Practices for neither NFT-R excreting dog: walking in the countryside around livestock; living with another animal except a cat,

Table S3. ST919 single-locus variant *E. coli* identified on Enterobase showing *nfsAB* mutation status and origins

Sample Accession	Source	Location	ST	*nfsAB* mutation?
SAMN46723104		United States	1865	N
SAMN45102163		United States	1865	N
SAMN44490190		United States	1865	N
SAMN43564816	Chicken Meat	Canada	919	Y
SAMN43563291	Chicken Meat	Canada	919	Y
SAMN43303518	Companion Animal	United States	919	N
SAMN41629489	Human	United Kingdom	919	N
SAMN40748560	Poultry	United States	1333	N
SAMN40748548	Chicken Meat	United States	919	Y
SAMN40737978	Companion Animal	United States	919	N
SAMN40372514	Wild Animal	Italy	1333	N
SAMN36864136	Companion Animal	United States	1333	N
SAMN32247434	Chicken Caecal Contents	Canada	919	Y
SAMN29767770		United States	1333	N
SAMN29598476	Companion Animal	United States	5328	N
SAMN29503068	Companion Animal	Canada	919	N
SAMN29042213	Poultry	United States	919	N
SAMN26305157	Human	United States	1333	N
SAMN25142514	Poultry	United States	919	N
SAMN20285927	Human	United Kingdom	1865	N
SAMN19949776	Wild Animal	United States	919	N
SAMN19069014	Wild Animal	United States	1333	N
SAMN14593721	Environment	United States	919	N
SAMN14530528	Chicken Caecal Contents	United States	919	Y
SAMN14530526	Chicken Caecal Contents	United States	919	Y
SAMN14530525	Chicken Caecal Contents	United States	919	Y
SAMN14530524	Chicken Caecal Contents	United States	919	Y
SAMN14530523	Chicken Caecal Contents	United States	919	Y
SAMN14530522	Chicken Caecal Contents	United States	919	Y
SAMN14113844	Environment	United States	919	N
SAMN14113834	Environment	United States	919	N
SAMN13721833	Poultry	United States	919	N
SAMN13513936	Environment	United States	919	N
SAMN13513935	Environment	United States	919	N
SAMN13513926	Environment	United States	10536	N
SAMN12359555	Poultry	United States	919	N
SAMN12241725	Chicken Yolk Sac Infection	Czechia	8874	Y
SAMN11444779	Chicken Caecal Contents	United Kingdom	919	Y
SAMN10875599	Human	Canada	919	N
SAMN10524481	Human	United Kingdom	1333	N
SAMN10396941	Environment	United States	1865	N
SAMN10221563	Poultry	United States	919	N
SAMN08039647	Environment		5328	N
SAMN08039645	Environment		5328	N
SAMN08039644	Environment		5328	N
SAMN07678444	Food	United States	919	N
SAMN07677607	Food	United States	919	N
SAMN07677496	Food	United States	919	N
SAMN05468056	Livestock	United States	919	N
SAMN05464555	Wild Animal	United States	1333	N
SAMN04992253	Livestock	United States	919	N
SAMEA7025515	Human UTI	United Kingdom	919	Y
SAMEA6163430	Human		1333	N
SAMEA4428396	Human	Netherlands	1333	N
SAMEA115667394	Human	Germany	1333	N
SAMEA115626452	Wild Animal	Denmark	919	N
SAMEA115372530		United Kingdom	919	N
SAMEA114461330			11823	N
SAMEA114461293			11823	N
SAMEA114461292			11823	N
SAMEA114461159			11823	N
SAMEA104437735		Poland	919	N
SAMEA104437717		Poland	919	N
SAMEA104314597			919	N
SAMEA104314596			919	N
SAMEA104314595			919	N
SAMEA104314589			1865	N
SAMEA104027456			919	N
SAMD00209584	Chicken Caecal Contents	Japan	919	Y
SAMD00209572	Chicken Caecal Contents	Japan	919	Y
SAMD00209571	Chicken Caecal Contents	Japan	919	Y

Table S4 SNP distance of ST919/ST8874 isolates carrying NfsA (212Stop) and NfsB (46Stop) loss-of-function mutations

		246497_ C1ANFT	254071_ RDFS15NFT	32816_ 10301NFTR	Reference	SAMD 00209584	SAMEA 7025515	SAMN 12241725	SAMN 14530522	SAMN 14530523	SAMN 14530525	SAMN 14530526	SAMN 32247434	SAMN 40748548	SAMN 43563291	SAMN 43564816
246497_C1ANFT	Bristol. Chicken ST8874	0	31	193	91	514	112	49	175	197	204	192	143	186	168	371
254071_RDFS15NFT	Bristol. RDF ST8874	31	0	202	100	523	121	58	184	206	213	201	152	195	177	380
32816_10301NFTR	Bristol. Dog ST919	193	202	0	172	533	193	180	176	196	199	181	146	49	73	276
SAMN11444779 (Reference)	UK. Chicken ST919	91	100	172	0	491	33	78	154	176	183	171	122	165	147	350
SAMD00209584*	Japan. Chicken ST919	514	523	533	491	0	512	501	557	565	572	552	525	528	510	696
SAMEA7025515	UK. Human UTI ST919	112	121	193	33	512	0	99	175	197	204	192	143	186	168	371
SAMN12241725	Czechia. Chicken ST8874	49	58	180	78	501	99	0	162	184	191	179	130	173	155	358
SAMN14530522	USA. Chicken ST919	175	184	176	154	557	175	162	0	108	115	107	106	169	151	352
SAMN14530523	USA. Chicken ST919	197	206	196	176	565	197	184	108	0	125	119	128	189	171	366
SAMN14530525	USA. Chicken ST919	204	213	199	183	572	204	191	115	125	0	128	135	192	174	375
SAMN14530526*	USA. Chicken ST919	192	201	181	171	552	192	179	107	119	128	0	123	174	156	353
SAMN32247434	Canada. Chicken ST919	143	152	146	122	525	143	130	106	128	135	123	0	139	121	324
SAMN40748548	USA. Chicken ST919	186	195	49	165	528	186	173	169	189	192	174	139	0	66	269
SAMN43563291	Canada. Chicken ST919	168	177	73	147	510	168	155	151	171	174	156	121	66	0	247
SAMN43564816	Canada. Chicken ST919	371	380	276	350	696	371	358	352	366	375	353	324	269	247	0

*These isolates are each representative of three identical isolates (Zero SNPs different)

Table S5. Nitrofurantoin pre-resistant ST665 *E. coli* identified on Enterobase with obvious loss of function mutations in *nfsA*

Accession	Country	Origin	NfsA variant	NfsB variant
SAMEA12944415	France	HUMAN	138FS	-
SAMEA26138668	Denmark	Animal Feed	138FS	-
SAMEA3484433	UK	Chicken Faeces	138FS	101Stop
SAMEA3484443	UK	Chicken Faeces	138FS	101Stop
SAMEA3484445	UK	Chicken Faeces	138FS	101Stop
SAMEA6471220	Portugal	POULTRY	138FS	-
SAMEA7281063	Netherlands	HUMAN	138FS	-
SAMEA8227248	Lithuania	POULTRY	138FS	-
SAMN09216415	Portugal	Wild Gull Faeces	138FS	-
SAMN14278962	Spain	POULTRY	138FS	-
SAMN15344973	Spain	HUMAN	138FS	-
SAMN16243148	Spain	White Stork	138FS	-
SAMN21845675	UK	HUMAN	138FS	101Stop
SAMN25649687	Spain	HUMAN	138FS	-
SAMN47554813	UK	HUMAN	138FS	101Stop
SAMEA13158773	Netherlands	HUMAN	46FS	-
SAMEA3752381	UK	Chicken Faeces	46FS	-
SAMEA5777946	Netherlands	Chicken Meat	46FS	-
SAMEA5777953	Netherlands	Chicken Meat	46FS	-
SAMN26427077	UK	Environment	46FS	-
SAMN46011680	France	HUMAN	46FS	-
SAMN09580040	Australia	Duck Faeces	45Stop	-
SAMN04279477	USA	Dog Faeces	49FS	-
SAMN11319457	Poland	HUMAN	49FS	-
SAMN14279003	Hungary	POULTRY	49FS	-
SAMN18120972	Lebanon	HUMAN	49FS	-
SAMN21214633	Italy	POULTRY	68FS	-
SAMN48012225	USA	Poultry	100Stop	-
SAMN07678428	USA	Unspecified Food	175FS	-
SAMN26427274	UK	Environment	-	35FS

Table S6. SNP distances between ST665 NFT-R/pre-resistant isolates with NfsA and NfsB (as stated) mutations

		246498_ C1CNFT	265798_ C1AT4	265831_ C3AT2	265853_ RDF6AMC1	SAMEA 5777946	SAMEA 5777953	SAMEA 13158773	Reference	SAMEA 12944415	SAMEA 26138668	SAMEA 3484433	SAMEA 3484443	SAMEA 3484445	SAMEA 3752381	SAMEA 6471220	SAMEA 7281063	SAMEA 8227248	SAMN 04279477	SAMN 09216415	SAMN 11319457	SAMN 14278962	SAMN 14279003	SAMN 15344973	SAMN 16243148	SAMN 18120972	SAMN 21845675	SAMN 25649687	SAMN 26427077	SAMN 46011680	SAMN 47554813
246498_ C1CNFT	Bristol Chicken 138FS 101Stop	0	2836	2969	2836	2818	2693	2824	3071	1966	2912	1776	1773	1774	2873	1943	2645	1966	2932	1972	2930	1968	2915	1921	1914	3498	2095	1920	3030	2817	2120
265798_ C1AT4	Bristol Chicken 46FS	2836	0	355	110	108	1861	84	2365	1856	3743	1844	1841	1842	553	1817	3095	1856	1849	1862	1852	1858	1830	2864	2857	2545	3129	2863	2001	105	3154
265831_ C3AT2	Bristol Chicken 46FS	2969	355	0	353	335	2088	341	2484	1991	3602	1975	1972	1973	268	1952	3230	1975	1973	1997	1973	1993	1951	2999	2992	2658	3260	2998	1768	334	3285
265853_ RDF6AMC1	Bristol RDF 46FS	2836	110	353	0	106	1861	96	2365	1854	3741	1842	1839	1840	551	1815	3093	1854	1849	1860	1852	1856	1830	2862	2855	2545	3127	2861	1999	105	3152
SAMEA 5777946	Netherlands Chicken 46FS	2818	108	335	106	0	1841	94	2347	1836	3723	1824	1821	1822	533	1797	3075	1836	1831	1842	1834	1838	1812	2844	2837	2527	3109	2843	1981	21	3134
SAMEA 5777953	Netherlands Chicken 46FS	2693	1861	2088	1861	1841	0	1849	2843	3203	4145	3191	3188	3189	2286	3164	3631	3203	2311	3209	2314	3205	2292	3133	3126	2829	3411	3132	2523	1840	3436
SAMEA 13158773	Netherlands Human 46FS	2824	84	341	96	94	1849	0	2353	1842	3729	1830	1827	1828	539	1803	3081	1842	1837	1848	1840	1844	1818	2850	2843	2533	3115	2849	1987	91	3140
Reference		3071	2365	2484	2365	2347	2843	2353	0	3204	4154	3192	3189	3190	2682	3165	3535	3132	2463	3210	2477	3206	2412	3234	3227	2909	3472	3233	2813	2346	3497
SAMEA 12944415	France Human 138FS	1966	1856	1991	1854	1836	3203	1842	3204	0	2150	212	209	210	1895	199	2232	212	2794	88	2792	84	2777	1314	1307	3420	2153	1313	2782	1835	2178
SAMEA 26138668	Denmark Animal Feed 138FS	2912	3743	3602	3741	3723	4145	3729	4154	2150	0	2139	2136	2137	3506	2110	3156	2135	3907	2156	3902	2152	3887	1810	1803	4446	2603	1809	3504	3722	2628
SAMEA 3484433	UK Chicken 138FS 101Stop	1776	1844	1975	1842	1824	3191	1830	3192	212	2139	0	15	16	1879	189	2220	212	2786	218	2784	214	2769	1302	1295	3412	1967	1301	2766	1823	1992
SAMEA 3484443	UK Chicken 138FS 101Stop	1773	1841	1972	1839	1821	3188	1827	3189	209	2136	15	0	9	1876	186	2217	209	2783	215	2781	211	2766	1299	1292	3409	1964	1298	2763	1820	1989
SAMEA 3484445	UK Chicken 138FS 101Stop	1774	1842	1973	1840	1822	3189	1828	3190	210	2137	16	9	0	1877	187	2218	210	2784	216	2782	212	2767	1300	1293	3410	1965	1299	2764	1821	1990
SAMEA 3752381	UK Chicken 46FS	2873	553	268	551	533	2286	539	2682	1895	3506	1879	1876	1877	0	1856	3134	1879	2171	1901	2171	1897	2149	2903	2896	2856	3164	2902	1966	532	3189
SAMEA 6471220	Portugal Chicken 138FS	1943	1817	1952	1815	1797	3164	1803	3165	199	2110	189	186	187	1856	0	2193	201	2757	205	2755	201	2740	1161	1154	3383	2130	1160	2743	1796	2155
SAMEA 7281063	Netherlands Human 138FS	2645	3095	3230	3093	3075	3631	3081	3535	2232	3156	2220	2217	2218	3134	2193	0	2232	3567	2238	3565	2234	3550	2152	2145	4132	1654	2151	3364	3074	1679
SAMEA 8227248	Lithuania Chicken 138FS	1966	1856	1975	1854	1836	3203	1842	3132	212	2135	212	209	210	1879	201	2232	0	2766	218	2724	214	2715	1316	1309	3361	2153	1315	2766	1835	2178
SAMN 04279477	USA Dog 49FS	2932	1849	1973	1849	1831	2311	1837	2463	2794	3907	2786	2783	2784	2171	2757	3567	2766	0	2800	262	2796	155	2995	2988	1976	3329	2994	2686	1830	3350
SAMN 09216415	Portugal Gull 138FS	1972	1862	1997	1860	1842	3209	1848	3210	88	2156	218	215	216	1901	205	2238	218	2800	0	2798	86	2783	1320	1313	3426	2159	1319	2788	1841	2184
SAMN 11319457	Poland Human 49FS	2930	1852	1973	1852	1834	2314	1840	2477	2792	3902	2784	2781	2782	2171	2755	3565	2724	262	2798	0	2794	207	2993	2986	2021	3327	2992	2686	1833	3352
SAMN 14278962	Spain Chicken 138FS	1968	1858	1993	1856	1838	3205	1844	3206	84	2152	214	211	212	1897	201	2234	214	2796	86	2794	0	2779	1316	1309	3422	2155	1315	2784	1837	2180
SAMN 14279003	Hungary Chicken 49FS	2915	1830	1951	1830	1812	2292	1818	2412	2777	3887	2769	2766	2767	2149	2740	3550	2715	155	2783	207	2779	0	2978	2971	1933	3312	2977	2664	1811	3337
SAMN 15344973	Spain Human 138FS	1921	2864	2999	2862	2844	3133	2850	3234	1314	1810	1302	1299	1300	2903	1161	2152	1316	2995	1320	2993	1316	2978	0	43	3452	1757	1	2842	2843	1782
SAMN 16243148	Spain Stork 138FS	1914	2857	2992	2855	2837	3126	2843	3227	1307	1803	1295	1292	1293	2896	1154	2145	1309	2988	1313	2986	1309	2971	43	0	3445	1750	42	2835	2836	1775
SAMN 18120972	Lebanon Human 49FS	3498	2545	2658	2545	2527	2829	2533	2909	3420	4446	3412	3409	3410	2856	3383	4132	3361	1976	3426	2021	3422	1933	3452	3445	0	3872	3451	3274	2526	3897
SAMN 21845675	UK Human 138FS 101Stop	2095	3129	3260	3127	3109	3411	3115	3472	2153	2603	1967	1964	1965	3164	2130	1654	2153	3329	2159	3327	2155	3312	1757	1750	3872	0	1756	3297	3108	59
SAMN 25649687	Spain Human 138FS	1920	2863	2998	2861	2843	3132	2849	3233	1313	1809	1301	1298	1299	2902	1160	2151	1315	2994	1319	2992	1315	2977	1	42	3451	1756	0	2841	2842	1781
SAMN 26427077	UK Environment 46FS	3030	2001	1768	1999	1981	2523	1987	2813	2782	3504	2766	2763	2764	1966	2743	3364	2766	2686	2788	2686	2784	2664	2842	2835	3274	3297	2841	0	1980	3322
SAMN 46011680	France Human 46FS	2817	105	334	105	21	1840	91	2346	1835	3722	1823	1820	1821	532	1796	3074	1835	1830	1841	1833	1837	1811	2843	2836	2526	3108	2842	1980	0	3133
SAMN 47554813	UK Human 138FS 101Stop	2120	3154	3285	3152	3134	3436	3140	3497	2178	2628	1992	1989	1990	3189	2155	1679	2178	3350	2184	3352	2180	3337	1782	1775	3897	59	1781	3322	3133	0

Table S7. Nitrofurantoin pre-resistant isolates from chicken meat and RDF in Bristol, United Kingdom, and relationships with human clinical isolates from Enterobase.

ST	Sample Type	NfsA Variant (all loss of function)	NfsB Variant	Human isolates with identical *nfsA* mutation (<150 SNPs)
10	Chicken Meat	226 Ins IS*1*	WT	None
57	Chicken Meat	M1I (ATG-ATA)	V93A	None
58	RDF	141 Ins IS*1*	M75I V93A	None
69	Chicken Meat (6 samples)	M1I	V93A	None
69	RDF	M1I	V93A	None
155	RDF	Del <150nt – 26	M75I V93A P209L	None
162	RDF	Del 109-110	M75I V93A A169T	None
665	Chicken Meat (2 samples)	46FS	M75I V93A	SAMEA13158773 (Netherlands), 84 SNPs SAMN46011680 (France), 105 SNPs
665	RDF	46FS	M75I V93A	SAMEA13158773 (Netherlands), 96 SNPs SAMN46011680 (France), 105 SNPs
752	Chicken Meat (5 samples)	M1I	WT	SAMN32868256 (UK), 43 SNPs SAMN25850013 (Netherlands), 61 SNPs SAMN14734242 (South Korean), 101 SNPs SAMN22183865 (UK), 135 SNPs
2509	RDF	44Stop	M75I V93A	None
2705	Chicken Meat	100Stop	M78I H80L V93A	None
7529	RDF (2 samples)	88Stop	M75I V93A	SAMN092904 (UK), 103 SNPs

Table S8 SNP distance of ST752 isolates

SampleID	Source	Country	Date	236996_C2Cspec	245209_C5Cspec	245223_C4Camox	265837_C3CT3	265839_C4AT1	265840_C4AT2	SAMN32235060	SAMN40442795	SAMN41381106	SAMN10840056	SAMEA7578064	SAMEA7578177	SAMEA8227255	SAMEA8227341	SAMEA12943667	SAMN11444790	SAMN32868256*	SAMN22183865	SAMN30900011	SAMN40231774	SAMN40497913	SAMN41449061	SAMN14734242	SAMN30416431	SAMEA3638314	SAMN25850018	SAMN25850013
SAMN32868256*	Human	United Kingdom	2023	2707	2785	43	2703	2709	1840	117	116	2004	2017	1147	48	2242	2343	109	2311	0	144	2015	3528	580	2602	110	2863	2588	3126	70
SAMN22183865	Human	United Kingdom	2021	2733	2811	135	2729	2735	1864	113	112	2002	2041	1171	122	2266	2365	105	2335	144	0	2039	3554	604	2630	144	2887	2610	3154	162
SAMN30900011	Human	United Kingdom	2022	2424	2508	2006	2426	2430	674	2012	2013	2535	52	2360	1993	2074	2403	2006	2281	2015	2039	0	3759	2190	3375	2017	2999	2811	3193	2033
SAMN40231774	Human	United Kingdom	2023	3360	3466	3519	3384	3390	3379	3529	3526	3398	3761	4165	3506	3242	3563	3521	3798	3528	3554	3759	0	3689	4886	3532	3918	1351	3929	3546
SAMN40497913	Human	United Kingdom	2024	2858	2936	571	2854	2860	2189	577	576	2111	2192	1273	558	2226	2314	569	2323	580	604	2190	3689	0	2706	580	2913	2755	3208	598
SAMN41449061	Human	United Kingdom	2023	3870	4006	2593	3924	3930	3442	2603	2602	3371	3377	3177	2580	3351	3494	2595	2733	2602	2630	3375	4886	2706	0	2603	4049	3979	4327	2620
SAMN14734242	Human	South Korea	2018	2709	2787	101	2705	2711	1842	117	116	2004	2019	1147	88	2242	2343	109	2311	110	144	2017	3532	580	2603	0	2863	2588	3128	126
SAMN30416431	Human	Ecuador	2021	3131	3199	2854	3117	3123	2962	2860	2859	2647	3001	2388	2841	3130	2583	2852	2834	2863	2887	2999	3918	2913	4049	2863	0	3052	3722	2881
SAMEA3638314	Human	Germany	2006	2426	2523	2579	2441	2447	2435	2585	2584	2572	2813	3221	2566	2295	2680	2577	3023	2588	2610	2811	1351	2755	3979	2588	3052	0	3052	2606
SAMN25850018	Human	Netherlands	2021	3377	3415	3117	3333	3339	3091	3127	3126	3338	3195	3773	3104	2938	3221	3119	3116	3126	3154	3193	3929	3208	4327	3128	3722	3052	0	3144
SAMN25850013	Human	Netherlands	2021	2725	2803	61	2721	2727	1858	135	132	2022	2035	1165	66	2260	2361	127	2329	70	162	2033	3546	598	2620	126	2881	2606	3144	0
SAMN32235060	Poultry	United States	2022	2704	2782	108	2700	2706	1837	0	47	1957	2014	1144	95	2239	2340	64	2306	117	113	2012	3529	577	2603	117	2860	2585	3127	135
SAMN40442795	Poultry	Mexico	2017	2705	2783	107	2701	2707	1838	47	0	1956	2015	1143	94	2238	2339	63	2307	116	112	2013	3526	576	2602	116	2859	2584	3126	132
SAMN41381106	Poultry	United States	2024	2843	2908	1995	2826	2832	2432	1957	1956	0	2537	2620	1982	2348	2126	1953	2353	2004	2002	2535	3398	2111	3371	2004	2647	2572	3338	2022
SAMN10840056	Poultry	United Kingdom	2015	2426	2510	2008	2428	2432	674	2014	2015	2537	0	2362	1995	2076	2405	2008	2283	2017	2041	52	3761	2192	3377	2019	3001	2813	3195	2035
SAMEA7578064	Poultry	Germany	2015	3111	3189	1138	3107	3113	2373	1144	1143	2620	2362	0	1125	2679	2856	1136	2761	1147	1171	2360	4165	1273	3177	1147	2388	3221	3773	1165
SAMEA7578177	Poultry	Denmark	2015	2685	2763	39	2681	2687	1818	95	94	1982	1995	1125	0	2220	2321	87	2289	48	122	1993	3506	558	2580	88	2841	2566	3104	66
SAMEA8227255	Poultry	Lithuania	2016	2665	2767	2233	2685	2693	2172	2239	2238	2348	2076	2679	2220	0	2479	2231	2289	2242	2266	2074	3242	2226	3351	2242	3130	2295	2938	2260
SAMEA8227341	Poultry	Spain	2016	2902	3044	2334	2962	2968	2479	2340	2339	2126	2405	2856	2321	2479	0	2332	2567	343	2365	2403	3563	2314	3494	2343	2583	2680	3221	2361
SAMEA12943667	Poultry	Canada	2015	2698	2776	100	2694	2700	1831	64	63	1953	2008	1136	87	2231	2332	0	2300	109	105	2006	3521	569	2595	109	2852	2577	3119	127
SAMN11444790	Poultry	United Kingdom	2017	2628	2770	2302	2688	2694	2345	2306	2307	2353	2283	2761	2289	2289	2567	2300	0	2311	2335	2281	3798	2323	2733	2311	2834	3023	3116	2329
236996_C2Cspec	Chicken meat	United Kingdom	2022	0	397	2698	329	345	2471	2704	2705	2843	2426	3111	2685	2665	2902	2698	2628	2707	2733	2424	3360	2858	3870	2709	3131	2426	3377	2725
245209_C5Cspec	Chicken meat	United Kingdom	2022	397	0	2776	154	170	2555	2782	2783	2908	2510	3189	2763	2767	3044	2776	2770	2785	2811	2508	3466	2936	4006	2787	3199	2523	3415	2803
245223_C4Camox	Chicken meat	United Kingdom	2022	2698	2776	0	2694	2700	1831	108	107	1995	2008	1138	39	2233	2334	100	2302	43	135	2006	3519	571	2593	101	2854	2579	3117	61
265837_C3CT3	Chicken meat	United Kingdom	2022	329	154	2694	0	88	2473	2700	2701	2826	2428	3107	2681	2685	2962	2694	2688	2703	2729	2426	3384	2854	3924	2705	3117	2441	3333	2721
265839_C4AT1	Chicken meat	United Kingdom	2022	345	170	2700	88	0	2479	2706	2707	2832	2432	3113	2687	2693	2968	2700	2694	2709	2735	2430	3390	2860	3930	2711	3123	2447	3339	2727
265840_C4AT2	Chicken meat	United Kingdom	2022	2471	2555	1831	2473	2479	0	1837	1838	2432	674	2373	1818	2172	2479	1831	2345	1840	1864	674	3379	2189	3442	1842	2962	2435	3091	1858
